# Genomic epidemiology of nosocomial carbapenemase-producing *Citrobacter freundii* in sewerage systems in the Helsinki metropolitan area, Finland

**DOI:** 10.3389/fmicb.2023.1165751

**Published:** 2023-05-26

**Authors:** Viivi Heljanko, Venla Johansson, Kati Räisänen, Veli-Jukka Anttila, Outi Lyytikäinen, Jari Jalava, Irma Weijo, Jaana-Marija Lehtinen, Kirsi-Maarit Lehto, Anssi Lipponen, Sami Oikarinen, Tarja Pitkänen, Annamari Heikinheimo, WastPan Study Group, Ahmad Al-Mustapha

**Affiliations:** ^1^Department of Food Hygiene and Environmental Health, Faculty of Veterinary Medicine, University of Helsinki, Helsinki, Finland; ^2^Department of Health Security, National Institute for Health and Welfare, Helsinki, Finland; ^3^Inflammation Center, Helsinki University Central Hospital, Helsinki, Finland; ^4^Faculty of Medicine, University of Helsinki, Helsinki, Finland; ^5^Faculty of Medicine and Health Technology, Tampere University, Tampere, Finland; ^6^Department of Health Security, National Institute for Health and Welfare, Kuopio, Finland; ^7^Finnish Food Authority, Ruokavirasto, Seinäjoki, Finland

**Keywords:** wastewater, wastewater surveillance, antimicrobial resistance, *Citrobacter freundii*, carbapenemase-producing *Citrobacter freundii*, carbapenemase-producing *Enterobacteriaceae*

## Abstract

Multi-drug resistance is emerging in *Citrobacter freundii*, which is the third most common carbapenemase-producing (CP) *Enterobacteriaceae* in humans in Finland due to recent outbreaks. The objective of this study was to determine if wastewater surveillance (WWS) could detect CP *C. freundii* strains causing infections in humans. Selective culturing was used to isolate CP *C. freundii* from the hospital environment, hospital wastewater, and untreated municipal wastewater in Helsinki, Finland, between 2019 and 2022. Species were identified using MALDI-TOF, and presumptive CP *C. freundii* isolates were subjected to antimicrobial susceptibility testing and further characterized by whole genome sequencing. A genomic comparison was conducted to compare isolates collected from the hospital environment, untreated municipal wastewater, and a selection of isolates from human specimens from two hospitals in the same city. We also examined the persistence of CP *C. freundii* in the hospital environment and the impact of our attempts to eradicate it. Overall, 27 *bla*_KPC − 2_-carrying *C. freundii* were detected in the hospital environment (ST18; *n* = 23 and ST8; *n* = 4), while 13 *bla*_KPC − 2_-carrying *C. freundii* (ST8) and five *bla*_VIM − 1_-carrying (ST421) *C. freundii* were identified in untreated municipal wastewater. CP *C. freundii* was not identified in hospital wastewater. We found three clusters (cluster distance threshold ≤ 10 allelic difference) after comparing the recovered isolates and a selection of isolates from human specimens. The first cluster consisted of ST18 isolates from the hospital environment (*n* = 23) and human specimens (*n* = 4), the second consisted of ST8 isolates from the hospital environment (*n* = 4), untreated municipal wastewater (*n* = 6), and human specimens (*n* = 2), and the third consisted of ST421 isolates from the untreated municipal wastewater (*n* = 5). Our results support previous studies suggesting that the hospital environment could act as a source of transmission of CP *C. freundii* in clinical settings. Furthermore, the eradication of CP Enterobacteriaceae from the hospital environment is challenging. Our findings also showed that CP *C. freundii* is persistent throughout the sewerage system and demonstrate the potential of WWS for detecting CP *C. freundii*.

## 1. Introduction

The World Health Organization has declared antimicrobial resistance (AMR) as one of the top 10 global public health threats, leading to increased mortality and a significant burden for healthcare and economies (WHO, 2021).

*Citrobacter* spp. are aerobic, Gram-negative, commensal bacteria commonly found within the gastrointestinal tract of humans and animals as well as in environmental sources such as soil and water (Forsythe et al., 2015). *Citrobacter* spp. can cause enteric diseases and extraintestinal infections such as infections of the urinary and respiratory tract. Less frequently, *Citrobacter* spp. also causes serious nosocomial infections in immunocompromised hosts (Forsythe et al., 2015). The occurrence of multi-drug resistance is increasing in *Citrobacters* that can carry extended-spectrum beta-lactamase (ESBL), AmpC beta-lactamases, and carbapenemase-encoding genes such as *bla*_KPC − 2_ (Zhang et al., 2008; Shahid, 2010).

From a hospital infection prevention viewpoint, *Citrobacter freundii* causes concerns due to its exceptional ability to accumulate resistance traits (Yao et al., 2021). The persistence of these strains in the hospital environment and their subsequent spread to patients can cause hard-to-treat infections in compromised hosts. In addition, the accumulated resistance traits could be re-transmitted beyond genus, family, and order boundaries (Majewski et al., 2017) to more virulent organisms such as *Klebsiella pneumoniae*. In the hospital environment, including water fixtures, such as sinks and toilets, affected patients are the suspected initial origin of the carbapenem-resistant organisms (Tofteland et al., 2013; Leitner et al., 2015). Microbes may persist in biofilms in water fixtures and then be dispersed to the surroundings by particles, droplets, or aerosol formation (Kotay et al., 2019). Indeed, it is challenging to verify the transmission of microbes to patients (Kizny Gordon et al., 2017). However, a significant relationship between contaminated sinks and the acquisition of carbapenemase-producing *Enterobacteriaceae* (CPE) in patients indicates the importance of hospital water fixtures and biofilms within them as the environmental reservoir of the CPE epidemics (Smolders et al., 2019). Studies showing hospital rooms as the epidemiological link in CPE outbreaks also support the role of the hospital environment as a source of CPE transmission (van Beek et al., 2019; Räisänen et al., 2021). Although total eradication of CPE in hospital water fixtures with cleaning or disinfection is shown to be difficult (Nurjadi et al., 2021), decreased colonization has been achieved by replacing the sinks and plumbing (Shaw et al., 2018; Smolders et al., 2019).

It has been proposed that wastewater surveillance (WWS) could be used for global surveillance, prediction, and early warning system of AMR (Hendriksen et al., 2019b). However, full integration of human, animal, and environmental AMR monitoring data is still missing. Moreover, there is a need for calibration and standardization of the different methods for reliable data comparison (Pruden et al., 2021). More research studies are needed to clarify the applicability of WWS in observing and predicting the clinically relevant AMR pathogens circulating in the community as well as within asymptomatic carriers. To the best of our knowledge, no previous studies have described the genomic epidemiology of carbapenemase-producing (CP) *C. freundii* in humans, hospitals, and untreated municipal wastewater.

In Finland, *C. freundii* is the third most common CPE in humans and one of the most common CPE-causing clusters (THL, 2022b). During 2012–2018, CP *C. freundii* represented 6.0% of CPE in human specimens (Räisänen et al., 2020), which increased rapidly to 20.0% in 2021 (THL, 2022b). Between 2016–2022, *bla*_KPC − 2_ CP *C. freundii* was detected in 60 human specimens in Finland.

This research was conducted to elaborate on the capacity of WWS to observe CP *C. freundii* strains causing infections. We assessed the similarity between the isolates from the hospital environment, untreated municipal wastewater, and a selection of isolates from human specimens from the national CPE strain collection, which is part of the national infectious diseases register sustained by the National Institute for Health and Welfare (THL). Additionally, we described the occurrence of CP *C. freundii* in the hospital environment and our attempts to eradicate it. The initial motivation for the sampling in the studied hospital arose when an unknown environmental transmission was suspected while one of the CP *C. freundii* clusters described by Räisänen et al. (2021) occurred in the studied hospital.

## 2. Materials and methods

### 2.1. Sample collection and bacterial isolation

**Figure 1:**
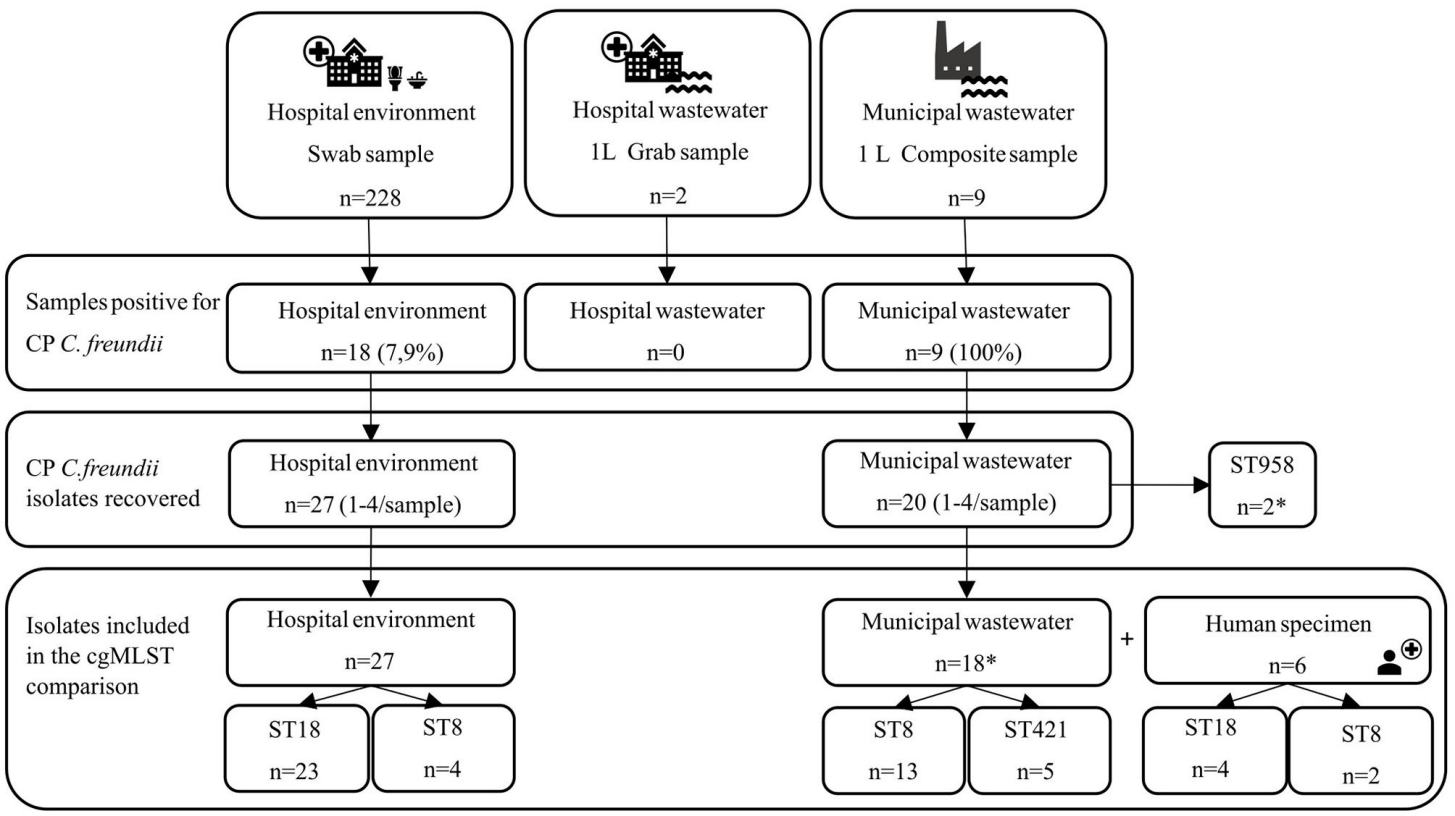
Illustration of the collected samples and recovered isolates. ST indicates sequence type. cgMLST indicates core genome multilocus sequence typing. Asterisk indicates two ST958 isolates that were left out of the cgMLST scheme due to poor sequence quality.

In this study, samples were collected from a hospital environment, with a specific focus on water fixtures such as sinks and toilets. Wastewater leaving from the same hospital to the municipal sewer was also sampled, as well as untreated municipal wastewater from a local wastewater treatment plant (WWTP) serving the area of the studied hospital (Figure 1). Sampling is described more precisely in the following paragraphs.

#### 2.1.1. Hospital environment

**Figure 2:**
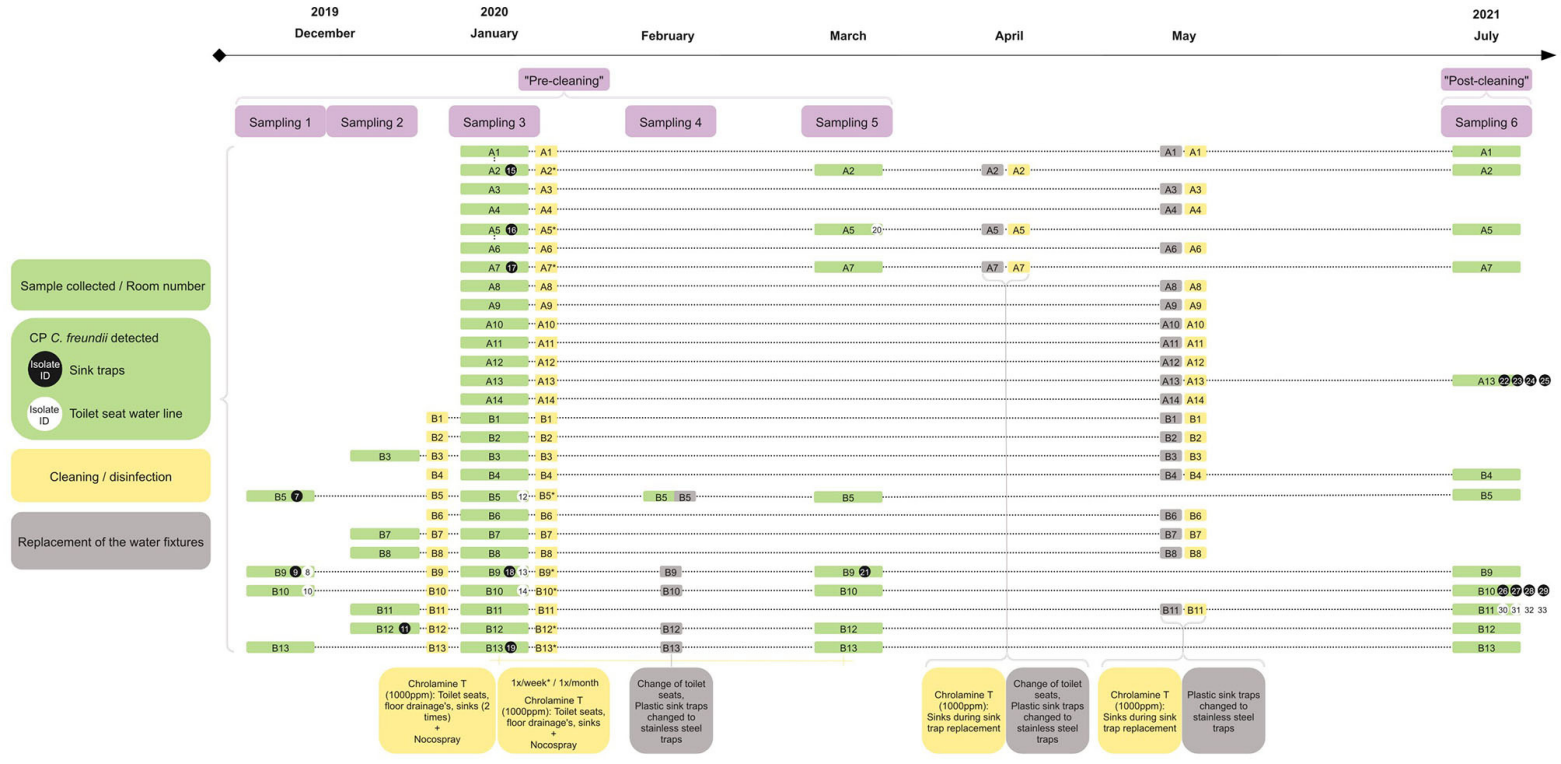
Illustration of the hospital environment sampling (purple rectangle) in hospital rooms (green rectangle) and detected carbapenemase-producing (CP) *Citrobacter freundii* isolates in sink traps (black circle) and toilets (white circle). The numbers inside or next to the circles indicate the isolate identification number (ID). Cleaning/disinfection (yellow rectangle) and replacement of the water fixtures (gray rectangle) are shown. Asterisk indicating rooms where cleaning and disinfection was performed a minimum of once per week after Sampling 3.

Sampling was conducted six times between December 2019 and July 2021, consisting of up to 27 patient rooms and 228 samples from the unit with two wards in one tertiary care hospital in Finland. The samples were obtained from sink traps (Supplementary Figure 1) and the toilet seat water line (Supplementary Figure 2). Additionally, samples were collected from sink taps (samplings 1–3), bidet showerhead and bidet shower sinks (samplings 1–2), toilet seat and sink plugholes, toilet seat water tank, and the cleaning and disinfection machine (sampling 4). Figure 2 illustrates the sampling timeline.

The samples obtained from the first five samplings (1–5) were collected before or during the cleaning actions (pre-cleaning). The samples were analyzed by THL following the protocol described by van Beek et al. (2019). The samples in sampling 6 (post-cleaning) were collected with sterile swabs (M40 Transystem Amies Agar Gel, Copan Diagnostics, Brescia, Italy) that were initially dampened with buffered peptone water (BPW) (Oxoid, Basingstoke, Hampshire, United Kingdom). The sampling site was scrubbed with the swab for 15–30 s, and then the swab was moved to the transfer tube. The samples were transferred to the University of Helsinki laboratory in a coolbox and further processed within 12 h from the time of collection.

Each cotton swab was placed into an Eppendorf tube with 1 ml of BPW. The tubes were vortexed, and 100 μl of the suspension was spread on CHROMagar mSuperCARBA (CHROMagar, Paris, France) and incubated aerobically for 18–24 h at 37°C. The remaining 900 μl of the BPW with the cotton swab was also incubated aerobically for 18–24 h at 37°C (enrichment) to detect small quantities of the targeted bacteria. After incubation, the enrichment was streaked with a 10 μl sterile loop on CHROMagar mSuperCARBA and incubated aerobically for 18–24 h at 37°C.

Colony morphology was observed according to the manufacturer's instructions. Overall, 1–5 typical metallic blue colonies were selected from each plate, refreshed with 1 μl sterile loop on CHROMagar mSuperCARBA, and incubated aerobically for 18–24 h at 37°C. Bacterial colonies were subcultured onto individual CHROMagar mSuperCARBA agar plates until a pure culture was achieved. Up to four isolates were subcultured on a bovine blood agar plate (Columbia Blood Agar Base, Oxoid Ltd., Basingstoke, United Kingdom) and incubated aerobically for 18–24 h at 37°C.

#### 2.1.2. Hospital wastewater

Two 1 L grab samples of wastewater from the hospital sewers were collected from two separate locations around the studied hospital. Sampling was performed in July 2021 in concordance with post-cleaning samples of the hospital environment. The samples were transferred to the laboratory in a coolbox and further processed within 12 h from the time of collection. A volume of 100 μl of the wastewater was pipetted and spread on CHROMagar mSuperCARBA and further processed as post-cleaning hospital environment samples without the enrichment step.

#### 2.1.3. Untreated municipal wastewater from local WWTP

Nine 24-h composite samples of untreated municipal wastewater were collected between February 2021 and February 2022 from the WWTP in the city. Wastewater was collected as a part of a consortium project “WastPan” (THL, 2022b). WWTP receives ~296,000 m^3^ of wastewater daily, and it serves ~800,000 inhabitants (14.5% of the population of Finland) (Tiwari et al., 2022). WWTP also receives wastewater from the studied hospital. A 1 L sample was delivered to the laboratory in a coolbox, from which 100 μl was pipetted and spread on CHROMagar mSuperCARBA and further processed as the post-cleaning hospital environment samples without the enrichment step.

### 2.2. Bacterial species identification

Isolates were identified using a matrix-assisted laser desorption ionization-time of flight mass spectrometry (MALDI-TOF MS) based Bruker Microflex LT/SH (Bruker Daltonics GmbH & Co. KG, Bremen, Germany) with a score value of >2.0, except for one isolate with a score value of 1.8, and the second-best match for *C. freundii* was identified with a score value of 1.7. Confirmed *C. freundii* isolates were stored at −80°C for further characterization.

### 2.3. Antimicrobial susceptibility testing

For presumptive CP *C. freundii* isolates, susceptibility to carbapenems was tested with meropenem (10 μg) (Abtek Biologicals Ltd., Liverpool, United Kingdom, or Rosco, Albertslund, Denmark) and ertapenem (10 μg) (Oxoid, Basingstoke, United Kingdom) with a disk diffusion test according to the European Committee of Antimicrobial Susceptibility Testing (EUCAST) standard (EUCAST, 2022a). The inhibition zones were measured with a digital caliper. Furthermore, an antimicrobial susceptibility testing was performed with the broth microdilution method using Sensititre EURGNCOL plates (Thermo Fischer Scientific, East Grinstead, United Kingdom) to determine the minimum inhibitory concentration (MIC) of colistin, piperacillin/tazobactam, ceftazidime/avibactam, ceftolozane/tazobactam, and meropenem. The method was performed according to the manufacturer's instructions, except for using 0.9% saline instead of sterile water. *Escherichia coli* ATCC 25922 was included as quality control for each Müeller-Hinton agar patch. The results were interpreted according to EUCAST epidemiological cut-off values (ECOFFs) (EUCAST, 2022b).

### 2.4. DNA extraction

Isolates were grown in tryptic soy broth (Oxoid, Basingstoke, UK) at 37°C for 16 h. DNA was extracted from cells harvested from 2 ml of culture using the QIAcube Connect instrument (QIAGEN, Hilden, Germany) with a DNeasy Blood & Tissue Kit (QIAGEN, Valencia, CA, United States). The quality of DNA was assessed by the A260/A280 ratio using a NanoDrop ND-1000 spectrophotometer (Thermo Fischer Scientific, Wilmington, DE, United States), and DNA quantity was measured using a Qubit 2.0 fluorometer (Invitrogen, Life Technologies, Carlsbad, CA, United States).

### 2.5. Whole genome sequencing and bioinformatical analyses

The extracted DNA from all 32 presumptive CP *C. freundii* isolates from the post-cleaning hospital environment (*n* = 12) and untreated municipal wastewater samples (*n* = 20) was outsourced for sequencing. Library preparation was performed with a NEBNext Ultra DNA Library Prep Kit for Illumina with 300 bp fragment length. Sequencing was performed with Illumina NovaSeq 6000 (Novogene, Cambridge, United Kingdom) with 2 × 150 bp read length and targeted genomic coverage of 100 ×.

Sequences were analyzed with Ridom SeqSphere+ software v7.7.5 (Ridom GmbH, Germany) (Jünemann et al., 2013). Quality analysis of the sequences was performed with FastQC v0.1.1.7 (Babraham Institute, 2021), and adapters were removed with Trimmomatic v0.36 (Bolger et al., 2014). Raw reads were assembled with SKESA v2.3.0 using default settings (Souvorov et al., 2018), and quality trimming was performed with an average quality of ≥30 and a window of 20 bases. Remapping and polishing were performed with the BWA-MEM mapping algorithm. Sequencing statistics are presented in Supplementary Table 1. AMR genes were identified from assembled genomes with NCBI AMRFinderPlus 3.2.3 (Feldgarden et al., 2019), using 100% alignment and >90% identity. ResFinder 4.1 (Center for Genomic Epidemiology web server, DTU, Denmark) (Zankari et al., 2017; Clausen et al., 2018; Bortolaia et al., 2020) was used with default settings to specify the allelic variants of beta-lactamase genes. Sequence types (ST) were analyzed using multilocus sequence type (MLST) (Becker et al., 2018) in Ridom SeqSphere+- (Ridom, Munster, Germany). Sequences with novel STs were submitted to PubMLST (Jolley et al., 2018) to assign new STs.

### 2.6. Genomic comparison of CP *C. freundii* isolates

All isolates, except for two from the untreated municipal wastewater with poor sequence quality, were included in an *ad hoc* core genome multilocus sequence type (cgMLST) scheme with CP *C. freundii* isolates from the pre-cleaning hospital environment samples (*n* = 15). Additionally, six isolates from human specimens from the national CPE strain collection (*n* = 6; ST18 4/48 and ST8 2/11) were included in the cgMLST scheme (Figure 1). The isolates from the human specimens with ST18 were obtained from inpatients treated in the studied unit and have been published previously by Räisänen et al. (2021). The isolates from the human specimens with ST8 were obtained from inpatients treated in another hospital in the same city and within the service area of the studied WWTP. Three isolates were obtained from clinical specimens (ST18, *n* = 2; ST8, *n* = 1) and two from screening specimens (ST18, *n* = 1; ST8, *n* = 1). The specimen type for one isolate was not specified (ST8, *n* = 1).

### 2.7. Cleaning and other interventions in the patient rooms of the studied hospital

After sampling rounds 1 and 2 in December 2019, the affected rooms were cleaned with 1,000 ppm Chloramine T (KiiltoClean, Finland). Additionally, undiluted Chloramine T was poured into the toilets, floor drainages (4.0 dL each), and sinks (1.5 dL). After a 1-h duration of action, rooms were disinfected with Nocospray (Oxy'Pharm, France). After sampling round 3 in January 2020, the cleaning routine continued as described above, but the patient rooms with CP *C. freundii* findings were cleaned at least once a week, and the other rooms at least once a month. The described routine was followed until the end of March 2020. Starting from February 2020, the toilet seats were replaced, and the plastic sink traps were changed to stainless steel traps (Figure 2).

## 3. Results

### 3.1. Hospital environment, hospital wastewater, and untreated municipal wastewater from local WWTP

The pre-cleaning hospital environment samples in December 2019 yielded four CP *C. freundii* isolates from three patient rooms (*n* = 3/4, 75.0%, sampling 1) and one isolate from one patient room (*n* = 1/5, 20.0%, sampling 2). In January 2020, eight CP *C. freundii* isolates were recovered from seven patient rooms (*n* = 7/27, 25.9%, sampling 3). In February 2020, CP *C. freundii* was not recovered from the samples (*n* = 0/5, 0.0%, sampling 4). In March 2020, sampling yielded two CP *C. freundii* isolates from two patient rooms (*n* = 2/8, 25.0%, sampling 5). Post-cleaning samples in July 2021 yielded 12 CP *C. freundii* isolates from three patient rooms (*n* = 3/12, 25.0%, sampling 6). Additional species identified from selective media in post-cleaning samples were *Klebsiella oxytoca* (*n* = 4) and *Klebsiella pneumoniae* (*n* = 2). Two isolates were not identifiable by MALDI-TOF. Figure 2 illustrates the sampling, findings, and interventions.

*C. freundii* was not recovered from the selective media from hospital wastewater samples (*n* = 0/2, 0%).

In total, 20 CP *C. freundii* isolates were recovered from nine untreated municipal wastewater samples (*n* = 9/9), with up to four CP *C. freundii* isolates in each sample.

### 3.2. Phenotypic susceptibility

The disk diffusion test and broth microdilution were performed for 32 *C. freundii* isolates recovered from the post-cleaning hospital environment (*n* = 12) and untreated municipal wastewater samples (*n* = 20). All isolates were considered to express phenotypical resistance against meropenem. The phenotypical susceptibility of the isolates is presented in Table 1.

**Table 1:** Zone of inhibition and MIC of antimicrobials for 32 *Citrobacter freundii* isolated from post-cleaning hospital environment sampling (HE, *n* = 12) and untreated municipal wastewater (WW, *n* = 20).

**Sampling site**	**Time (MM/YY)**	**Isolate ID**	**Zone of inhibition (mm)**		**MIC (mg/L)**				
			**MRP10**	**ERT10**	**COL**	**P/T4**	**C/T**	**CZA**	**MERO**
(ECOFFs)			25	N/A	I/D	8	1	N/A	I/D
HE	07/21	22	**18.3**	17.7	0.5	**>32**	**>8**	1	4
	07/21	23	**18.3**	19	0.5	**>32**	**>8**	1	2
	07/21	24	**19.0**	19.8	0.5	**>32**	**8**	1	2
	07/21	25	**19.7**	18.8	0.5	**>32**	**>8**	1	4
	07/21	26	**22.9**	20.6	0.5	**>32**	**>8**	1	2
	07/21	27	**23.0**	19.9	0.5	**>32**	**>8**	1	2
	07/21	28	**24.2**	21.6	0.5	**>32**	**8**	1	2
	07/21	29	**22.7**	20.2	0.5	**>32**	**>8**	1	2
	07/21	30	**22.0**	19.7	0.5	**>32**	**>8**	1	4
	07/21	31	**24.7**	19.4	0.5	**>32**	**>8**	1	2
	07/21	32	**23.6**	19.9	0.5	**>32**	**>8**	1	4
	07/21	33	**21.6**	18.3	0.5	**>32**	**>8**	1	2
WW	02/21	34	**24.5** ^*^	21.1	0.5	**>32**	**1**	< 1	0.5
	02/21	35	**18.6** ^*^	17.9	0.5	**>32**	**8**	< 1	8
	02/21	36	**16.2** ^*^	18.6	< 0.25	**>32**	**2**	< 1	8
	04/21	37	**16.5** ^*^	17.1	< 0.25	**>32**	**8**	< 1	8
	05/21	38	**19.0** ^*^	20.0	< 0.25	**>32**	**4**	< 1	8
	05/21	39	**17.0** ^*^	17.0	< 0.25	**>32**	**4**	< 1	8
	07/21	40	**14.4**	18.0	0.5	**>32**	**8**	< 1	8
	07/21	41	**15.5**	15.9	0.5	**>32**	**>8**	< 1	4
	07/21	42	**17.2**	19.0	0.5	**>32**	**4**	< 1	4
	08/21	43	**23.8** ^*^	17.0	0.5	**>32**	**1**	< 1	1
	08/21	44	**15.7** ^*^	14.4	0.5	**>32**	**>8**	< 1	8
	10/21	45	**21.1** ^*^	18.6	0.5	**>32**	**4**	< 1	8
	10/21	46	**22.8** ^*^	22.2	0.5	**>32**	**8**	< 1	8
	11/21	47	**18.4** ^*^	24.4	0.5	**>32**	**>8**	< 1	4
	11/21	48	**18.9** ^*^	23.0	0.5	**>32**	**>8**	< 1	4
	01/22	49	**15.7** ^*^	21.2	0.5	**>32**	**>8**	< 1	8
	02/22	50	**6.0** ^*^	7.5	0.5	**>32**	**>8**	8	>16
	02/22	51	**16.1** ^*^	23.0	0.5	**>32**	**>8**	< 1	4
	02/22	52	**14.0** ^*^	18.6	0.5	**>32**	**4**	< 1	8
	02/22	53	**13.0** ^*^	16.8	0.5	**>32**	**8**	< 1	8

Epidemiological cut-off values (ECOFFs) (mm and mg/L) are indicated. N/A indicates that ECOFF is not applicable. I/D indicates insufficient data. Bold lettering indicates Zone of inhibition below and MICs above the ECOFF. MRP10 Meropenem (10 μg). ERT10 Ertapenem (10 μg). Meropenem disk 10 μg by Abtek Biologicals Ltd. (Liverpool, United Kingdom) indicated by ^*^ and by Rosco (Albertslund, Denmark) without indicator. COL Colistin, P/T4 Piperacillin/Tazobactam, C/T Ceftolozane/Tazobactam, CZA Ceftazidime/Avibactam, and MERO Meropenem.

### 3.3. Whole genome sequencing and bioinformatic analyses

In total, four different STs of CP *C. freundii* were identified. In the post-cleaning hospital environment samples, the most prevalent was ST18 (*n* = 8/12, 66.7%), followed by ST8 (*n* = 4/12, 33.3%). In the untreated municipal wastewater, the most prevalent was ST8 (*n* = 13/20, 65.0%), followed by ST421 (*n* = 5/20, 25.0%) and ST958 (*n* = 2/20, 10.0%) (Table 2). All the pre-cleaning hospital environment samples included in the genomic comparison belonged to ST18 (*n* = 15), and the isolates from human specimens included in the comparison belonged to ST18 (*n* = 4) and ST8 (*n* = 2) (Figure 1).

**Table 2:** Genomic characteristics for the 32 *Citrobacter freundii* isolated from post-cleaning hospital environment sampling (HE, *n* = 12) and untreated municipal wastewater (WW, *n* = 20).

			**Resistance genes**		
**Sampling site**	**Isolate ID**	**MLST**	**Carbapenemase genes**	**Other** ***bla*** **genes**	**Resistance genes other than** ***bla***
HE	22	ST8	*bla*_KPC − 2_	*bla*_OXA − 9_,*bla*_CMY − 79_,*bla*_CMY − 116_, *bla*_TEM − 1A_	*aac(6′)-If, aph(6)-Id, aac(3)-IIa, aadA1, dfrA1, sul2, sat2*
	23	ST8	*bla*_KPC − 2_	*bla*_OXA − 9_,*bla*_CMY − 79_,*bla*_CMY − 116_, *bla*_TEM − 1A_	*aac(6′)-If, aph(6)-Id, aac(3)-IIa, aadA1, dfrA1, sul2, sat2*
	24	ST8	*bla*_KPC − 2_	*bla*_OXA − 9_,*bla*_CMY − 79_,*bla*_CMY − 116_, *bla*_TEM − 1A_	*aac(6′)-If, aph(6)-Id, aac(3)-IIa, aadA1, dfrA1, sul2, sat2*
	25	ST8	*bla*_KPC − 2_	*bla*_OXA − 9_,*bla*_CMY − 79_,*bla*_CMY − 116_, *bla*_TEM − 1A_	*aac(6′)-If, aph(6)-Id, aac(3)-IIa, aadA1, dfrA1, sul2, sat2*
	26	ST18	*bla*_KPC − 2_	*bla*_OXA − 9_,*bla*_CMY − 117_,$bla_{\text{TEM}-\text{XX}}^{*}$	*aph(6)-Id, aac(3)-IIa, aadA5, dfrA17*
	27	ST18	*bla*_KPC − 2_	*bla*_OXA − 9_,*bla*_CMY − 117_,$bla_{\text{TEM}-\text{XX}}^{*}$	*aph(6)-Id, aac(3)-IIa, aadA5, dfrA17*
	28	ST18	*bla*_KPC − 2_	*bla*_OXA − 9_,*bla*_CMY − 117_,$bla_{\text{TEM}-\text{XX}}^{*}$	*aph(6)-Id, aac(3)-IIa, aadA5, dfrA17*
	29	ST18	*bla*_KPC − 2_	*bla*_OXA − 9_,*bla*_CMY − 117_,$bla_{\text{TEM}-\text{XX}}^{*}$	*aph(6)-Id, aac(3)-IIa, aadA5, dfrA17*
	30	ST18	*bla*_KPC − 2_	*bla*_OXA − 9_,*bla*_CMY − 117_,$bla_{\text{TEM}-\text{XX}}^{*}$	*aph(6)-Id, aac(3)-IIa, aadA5, dfrA17*
	31	ST18	*bla*_KPC − 2_	*bla*_OXA − 9_,*bla*_CMY − 117_,$bla_{\text{TEM}-\text{XX}}^{*}$	*aph(6)-Id, aac(3)-IIa aadA5, dfrA17*
	32	ST18	*bla*_KPC − 2_	*bla*_OXA − 9_,*bla*_CMY − 117_,$bla_{\text{TEM}-\text{XX}}^{*}$	*aph(6)-Id, aac(3)-IIa, aadA5, dfrA17*
	33	ST18	*bla*_KPC − 2_	*bla*_OXA − 9_,*bla*_CMY − 117_,$bla_{\text{TEM}-\text{XX}}^{*}$	*aph(6)-Id, aac(3)-IIa, aadA5, dfrA17*
WW	34^1^	ST958	NF	*bla*_OXA − 10_,*bla*_CFE_, $bla_{\text{CMY}-100}^{*}$	*qnrB, cmlA5*
	35	ST8	*bla*_KPC − 2_	*bla*_CMY − 79_,*bla*_CMY − 116_,$bla_{\text{TEM}-\text{XX}}^{*}$	*aac(6′)-If, aadA1, dfrA1, sul2, sat2*
	36	ST8	*bla*_KPC − 2_	*bla*_CMY − 79_,*bla*_CMY − 116_,$bla_{\text{TEM}-\text{XX}}^{*}$	*aac(6′)-If, aadA1, dfrA1, sul2, sat2*
	37	ST8	*bla*_KPC − 2_	*bla*_CMY − 79_,*bla*_CMY − 116_,$bla_{\text{TEM}-\text{XX}}^{*}$	*aac(6′)-If, aadA1, dfrA1, sul2, sat2*
	38	ST8	*bla*_KPC − 2_	*bla*_CMY − 79_,*bla*_CMY − 116_,$bla_{\text{TEM}-\text{XX}}^{*}$	*aac(6′)-If, aadA1, dfrA1, sul2, sat2*
	39	ST8	*bla*_KPC − 2_	*bla*_CMY − 79_,*bla*_CMY − 116_,$bla_{\text{TEM}-\text{XX}}^{*}$	*aac(6′)-If, aadA1, dfrA1, sul2, sat2*
	40	ST8	*bla*_KPC − 2_	*bla*_CMY − 79_,*bla*_CMY − 116_,$bla_{\text{TEM}-\text{XX}}^{*}$	*aac(6′)-If, aadA1, dfrA1, sul2, sat2*
	41	ST8	*bla*_KPC − 2_	*bla*_CMY − 79_,*bla*_CMY − 116_,$bla_{\text{TEM}-\text{XX}}^{*}$	*aac(6′)-If, aadA1, dfrA1, sul2, sat2*
	42	ST8	*bla*_KPC − 2_	*bla*_CMY − 79_,*bla*_CMY − 116_,$bla_{\text{TEM}-\text{XX}}^{*}$	*aac(6′)-If, aadA1, dfrA1, sul2, sat2*
	43^1^	ST958	NF	*bla*_OXA − 10_,*bla*_CFE_, $bla_{\text{CMY}-100}^{*}$	*aac(6′)-Ib, aadA2, sul1, qnrB, cmlA5*
	44	ST421	*bla*_VIM − 1_	*bla*_CMY − 40_, *bla*_CTX − M−15_	*aac(6′)-Iic, dfrA16, sul1, qnrS1, qnrB9, mph*(E)*, msr*(E)
	45	ST8	*bla*_KPC − 2_	*bla*_CMY − 79_, *bla*_CMY − 116_, $bla_{\text{TEM}-1\text{B}}^{*}$	*aac(6′)-If, aadA1, dfrA1, sul2, sat2*
	46	ST8	*bla*_KPC − 2_	*bla*_CMY − 79_, *bla*_CMY − 116_, *bla*_TEM − 1_	*aac(6′)-If, aadA1, dfrA1, sul2, sat2*
	47	ST421	*bla*_VIM − 1_	*bla*_CMY − 40_, *bla*_CTX − M−15_	*aac(6′)-IIc, dfrA16, sul1, qnrS1, qnrB9*
	48	ST421	*bla*_VIM − 1_	*bla*_CMY − 40_, *bla*_CTX − M−15_	*aac(6′)-IIc, dfrA16, sul1, qnrS1, qnrB9, mph*(E)*, msr*(E)
	49	ST421	*bla*_VIM − 1_	*bla*_CMY − 40_, *bla*_CTX − M−15_	*aac(6′)-IIc, dfrA16, sul1, qnrS1, qnrB9, mph*(E)*, msr*(E)
	50	ST8	*bla*_KPC − 2_	*bla*_CMY − 79_, *bla*_CMY − 116_, $bla_{\text{TEM}-\text{XX}}^{*}$	*aac(6′)-If, aph(3′')-Ib, aph(6)-Id, aadA1, dfrA1, sul2, sat2, qnrS1*
	51	ST421	*bla*_VIM − 1_	*bla*_CMY − 40_, *bla*_CTX − M−15_	*aac(6′)-IIc, dfrA16, sul1, qnrS1, qnrB9, mph*(E)*, msr*(E)
	52	ST8	*bla*_KPC − 2_	*bla*_CMY − 79_, *bla*_CMY − 116_	*aac(6′)-If, aadA1, dfrA1, sul2, sat2*
	53	ST8	*bla*_KPC − 2_	*bla*_CMY − 79_, *bla*_CMY − 116_	*aac(6′)-If, aadA1, dfrA1, sul2, sat2*

MLST, Multi-locus Sequence Type; NF, Not found. AMRfinderPlus v.3.2.3 for antimicrobial resistance genes and ResFinder 4.1. to determine the allelic variants of beta-lactamase genes (*bla*). Beta-lactamase gene detected only by ResFinder 4.1. indicated with ^*^. *bla*_TEM − XX_ indicates an unidentified TEM-variant due to low alignment. (1) Sequences (*n* = 2) did not exceed the quality requirements for the core genome multi-locus sequence type scheme.

All isolates (*n* = 27) from the hospital environment carried the *bla*_KPC − 2_ carbapenemase gene. *bla*_KPC − 2_ was also the most prevalent carbapenemase gene in isolates observed in the untreated municipal wastewater (*n* = 13/20, 65.0%), followed by *bla*_VIM − 1_ (*n* = 5/20, 25.0%). Carbapenemase genes could not be identified in two isolates from untreated municipal wastewater (*n* = 2/20, 10.0%). Additionally, 4 to 12 other AMR genes were identified from each isolate. All identified AMR genes in the post-cleaning hospital environment and untreated municipal wastewater isolates are presented in Table 2.

### 3.4. Genomic comparison of CP *C. freundii* isolates

**Figure 3:**
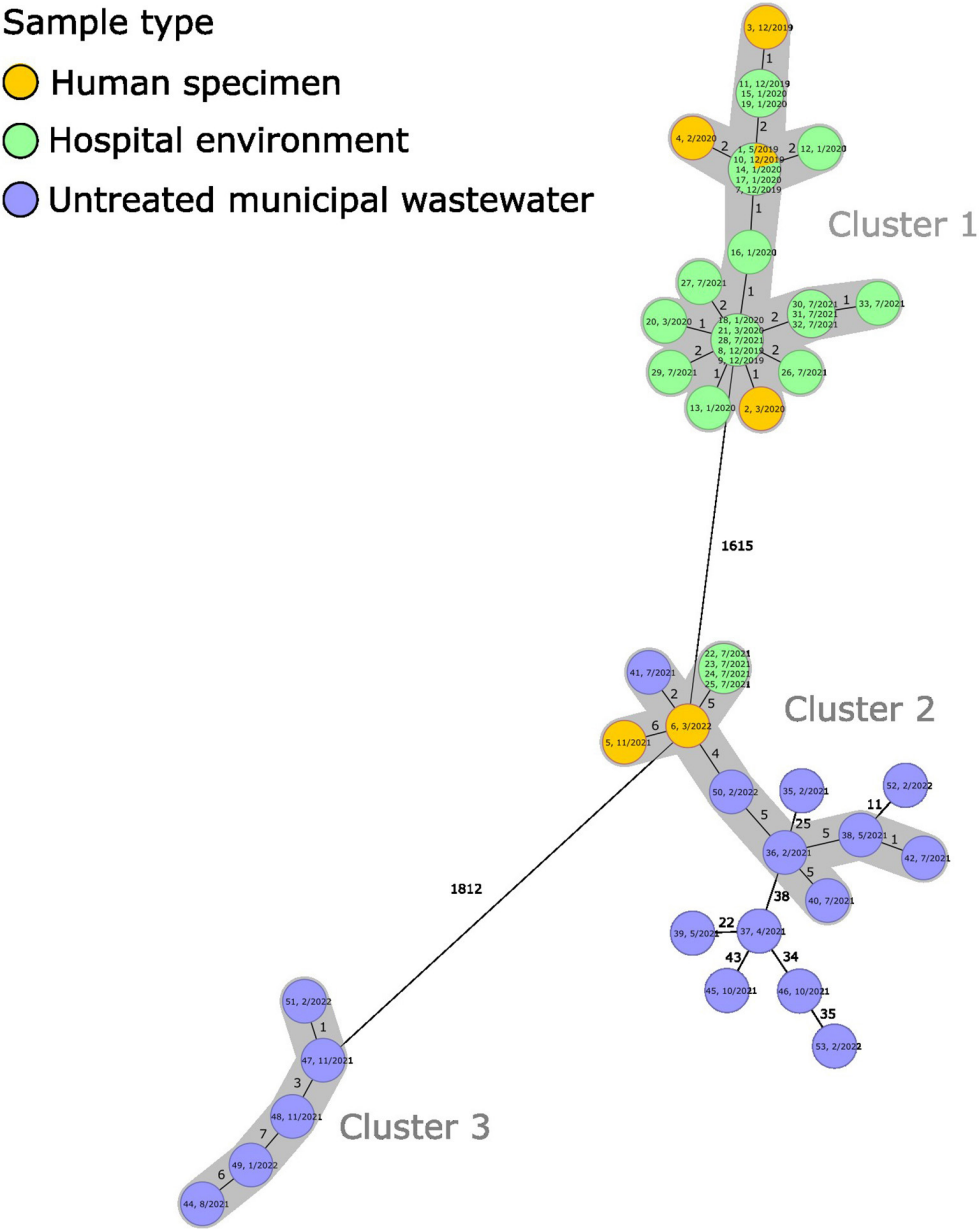
A minimum spanning tree of *ad hoc* core genome multilocus sequence typing (cgMLST) of 51 carbapenemase-producing *Citrobacter freundii* isolates, forming clusters 1 (ST18), 2 (ST8), and 3 (ST421). Each circle represents one or multiple identical sequences, and the numbers between the circles indicate the allele differences. Text in the circle indicates the isolate identification number and sample month/year; colors indicate the sample type [human specimen (yellow), hospital environment (green), and untreated municipal wastewater (blue)]. Human specimen isolates (1–6) originated from the national CPE strain collection and were obtained from clinical specimens (isolates 1, 2, and 5), screening specimens (isolates 3 and 4), and an unspecified sample type (isolate 6). Isolates 1–4 have been published previously by Räisänen et al. (2021). The seed genome was NZ_CP007557.1, and the cgMLST was based on 2007 targets, pairwise ignoring missing values.

We identified three clusters [cluster threshold ≤ 10 allele difference (Jamin et al., 2021)] shown in Figure 3. Cluster 1 comprised isolates belonging to ST18— 23 from the hospital environment and 4 from the human specimens. Cluster 2 comprised isolates belonging to ST8, from which, four, six, and two originated from the hospital environment, untreated municipal wastewater, and human specimens, respectively. Cluster 3 comprised five isolates belonging to ST421 from untreated municipal wastewater. Clusters 1, 2, and 3 were separated by a long distance (up to 1812 allele difference) (Figure 3).

## 4. Discussion

In this study, we identified closely related CP *C. freundii* strains belonging to ST8 in the hospital environment, untreated municipal wastewater, and human specimens, demonstrating that CP *C. freundii* strains causing infections can be detected in untreated municipal wastewater. However, the absence of ST18 in untreated municipal wastewater raises questions about the sensitivity of culture-based WWS to detect all clinically relevant strains. ST18 has been the dominant ST in human specimens, responsible for an ongoing outbreak since 2016 (Räisänen et al., 2021), whereas ST8 has been rarer.

In this study, we collected the municipal wastewater samples in 2021–2022, whereas the human isolates included in the comparison were from 2019–2020 (ST18) and 2021–2022 (ST8). ST18 was first discovered in a human specimen in 2016 in one hospital located in the same region. The temporal variance between the samples should be noted when interpreting the results. Accordingly, undetected ST18 in municipal wastewater could reflect that it was less frequent in the population during the municipal wastewater sampling, whereas ST8 could have been more common than realized. Hundreds of screening specimens collected from the patients treated in the unit of the studied hospital in 2019 showed a low prevalence of CP *C. freundii* (data not published). However, the true prevalence of asymptomatic carriage in the Finnish population is unknown. Regarding the above, asymptomatic carriage of CP *C. freundii* ST8 may be more common than recognized and, in combination with a slight increase of detected CP *C. freundii* ST8 in clinical specimens in Finland recently, could be reflected in the number of ST8 isolates derived from untreated municipal wastewater. Moreover, a clonal shift in the bacterial population (Shapiro, 2016) could also explain why ST18 could not be recovered from wastewater. *C. freundii* is a commensal bacterium carried within the gastrointestinal tract of both humans and animals (Forsythe et al., 2015). In this study, the animal origin of CP *C. freundii* in untreated municipal wastewater is unlikely, as CPE has never been reported in food producing animals in Finland in routinely performed national surveillance (Finnish Food Authority, 2022); only one case of CP *Escherichia coli* has been reported in companion animals in Finland (Grönthal et al., 2018). However, several studies have reported CPE in wildlife, including wild boars in Algeria (Bachiri et al., 2018) and wild avian species in Spain (Oteo et al., 2018). CPE has not been detected in migratory barnacle geese in Finland (Kurittu et al., 2021), but we cannot comprehensively exclude the possibility of the wildlife origin of CP *C. freundii*, as research data related to CPE in wildlife in Finland is otherwise scarce.

Undetected ST18 in untreated municipal wastewater may indicate its possibly shorter survival time in the wastewater matrix, leading to a quick depletion. In *E. coli*, different strains have shown substantial differences in growth under the same growth conditions (Sekse et al., 2012). CP *C. freundii* belonging to ST8 may have a longer survival time in wastewater, allowing it to survive better in the conditions of the WWTP. ST8 may also be predominant or endemic in the sewerage network or studied WWTP, and isolates may reflect the established microbiome of the sewerage network rather than that of the entire population (LaMartina et al., 2021). The enormous microbial load and a favorable environment in wastewater promote bacterial growth, biofilm formation (Weingarten et al., 2018), and sharing of mobile genetic elements—Even beyond genus, family, and order boundaries (Majewski et al., 2017). All these factors could lead to differences in the AMR bacteria detected in the municipal wastewater compared to those circulating in the population.

CP *C. freundii* was not detected in hospital wastewater, most likely due to the chosen sampling method. A grab sample represents the present situation during the sample collection. Hence, the sample can represent anything from toilet water to gray water, such as showering or cleaning water, leading to substantial differences in the microbiome of that particular sample. A 24-h composite sample would be more representative and less prone to the effects of individual events. Hospital wastewater may also contain greater concentrations of cleaning or disinfection agents that influence the viability of bacteria, and molecular methods could be less sensitive to these effects (Girones et al., 2010).

The genetic relatedness of CP *C. freundii* isolates from the hospital environment, untreated municipal wastewater, and human specimens indicates a possible persistence of the bacteria throughout the sewerage system and a passage of this microbe from patients or the hospital environment to the WWTP. However, we cannot comprehensively prove the directionality, hence further studies are needed to verify this hypothesis. The genetic relatedness identified between the CP *C. freundii* ST18 isolates from the hospital environment and human specimens supports the previous hypothesis of a possible environmental source of transmission of this microbe (Räisänen et al., 2021). Sinks and toilets in hospitals may facilitate the storage of human-origin strains (Nurjadi et al., 2021) in biofilms and form a rich ecosystem where plasmids carrying carbapenemase-encoding genes could be transferred through horizontal gene transfer between bacteria (Majewski et al., 2017). Epidemics in hospitals can arise from the patients, but the hospital environment may also facilitate a possible source of transmission of CP *C. freundii* in clinical settings.

CP *C. freundii* was detected both in sink traps and toilets in the studied hospital, but not in the other sampling sites (sink taps, bidet showerhead and bidet shower sinks, toilet seat and sink plugholes, toilet seat water tank, or cleaning and disinfection machine). These findings are in line with the studies that have shown drains to be the most common reservoirs of CPE in the hospital environment (Park et al., 2020; Nurjadi et al., 2021). We observed recurrent findings with clonal isolates in four patient rooms throughout the study, despite regular treatment with Chloramine T (1,000 ppm) and the replacement of the toilet seats and sink traps (Figure 2). The clonality of the detected isolates reflects the persistence of these strains in the water fixtures rather than the recolonization caused by the patients. Previous studies have shown that cleaning and disinfection reduce the bacterial load, but a total eradication of CPE in hospital water fixtures is rarely achieved (Hopman et al., 2019; Nurjadi et al., 2021) and re-emergence is common despite intensive cleaning or replacement of the water fixtures (Decraene et al., 2018; Shaw et al., 2018; Smolders et al., 2019). Constructional interventions are the only reported successful measures to contain outbreaks (Nurjadi et al., 2021). It is essential to ensure that the surfaces in the hospital environment are easy to keep clean, but more research studies are needed to develop effective eradication methods and technologies that prevent the spread of microbes from sinks and toilets to the surroundings.

A culture-based approach, combined with whole genome sequencing, was chosen to evaluate the convenience of the commercially available culture media in this context. Molecular methods, such as metagenomic sequencing, may provide deeper taxonomic profiles and give a broad overview of the resistance genes. However, metagenomic sequencing may struggle to identify species at the strain level and assign detected resistance genes to particular species (Hendriksen et al., 2019a). Metagenomic sequencing is also expensive to perform and requires expertise that is commonly lacking at the local level (Pruden et al., 2021). Targeted PCR can detect resistance genes in the wastewater (Pärnänen et al., 2019; Probst et al., 2022) but is limited by the target panel and does not provide information about the species carrying the resistance genes (Girones et al., 2010). Pruden et al. (2021) stated that a culture-based approach is attractive since fecal indicator monitoring infrastructure is relatively low cost and already widespread and accessible in many low and low-middle-income countries. However, the culture-based approach has its limitations. For example, not all bacteria are culturable (Stewart, 2012), and the culture media can significantly impact the recovery of the bacteria from the samples (Davis et al., 2005). Nevertheless, culture-based methods can be as sensitive, specific, and accurate as PCR-based methods if the target organism is culturable (Bliem et al., 2018), as is the case with *Enterobacteriaceae*, which are commonly uncomplicated to recover from samples. CHROMagar mSuperCARBA has shown enough sensitivity and specificity to detect CPE in clinical specimens (Soria Segarra et al., 2018). However, wastewater is a complex sample material, and identification of the species of interest by colony morphology on selective media can be difficult. The spectrum of species is much broader than in clinical specimens, and environmental microbes may disturb species identification. In our study, we could only choose a pre-agreed number of colonies from the untreated municipal wastewater samples, and the manual selection of colonies can, by chance, lead to the discarding of some bacterial species or isolates with different STs and enzyme types. The apparent advantage—but also limitation—of culture-based WWS is that the analyses give information about a single bacterial clone. The clones can be described precisely and compared with the clones from various sources. However, studying all relevant clones requires extensive culturing and whole genome sequencing, which is highly laborious and increases costs.

Clinical surveillance is undoubtedly essential in screening and observing AMR pathogens in patients at a higher risk. However, it seems that, despite its limitations, culture-based WWS has the potential to detect AMR pathogens causing infections in the population and possibly also in detecting asymptomatic circulation at the population level. Additionally, WWS is decisive in identifying possible new threats, the persistence or emergence of AMR genes, and following the AMR trends. Research on WWS of AMR still needs to contribute to answering many questions, from standardized methodology to integration of existing AMR data. Longitudinal surveillance, including clinical and wastewater samples, and optimally also data from an extensive screening of the healthy Finnish population, could reveal whether the dominance of ST8 in the wastewater is indicative of its incidence in the population, predicted its elevation in the clinical specimens in Finland, or reflected the microbiome of the WWTP. WWS should not be seen as a competitor or a replacement for clinical surveillance, but as a tool that can provide a broader view of the AMR situation in the population. In the future, the insights provided by WWS would be helpful also in clinical settings and decision-making.

## Data availability statement

The datasets presented in this study can be found in online repositories. The names of the repository/repositories and accession number(s) can be found below: https://www.ebi.ac.uk/ena, PRJEB58690.

## Author contributions

VH: conceptualization, methodology, formal analysis, investigation, data curation, writing—original draft, writing—review and editing, and visualization. VJ: conceptualization, methodology, investigation, and writing—review and editing. KR: conceptualization, formal analysis, resources, writing—review and editing, and visualization. V-JA and OL: conceptualization, resources, and writing—review and editing. JJ, IW, and J-ML: resources and writing—review and editing. K-ML, AL, SO, and TP: writing—review and editing and project administration. AH: conceptualization, writing—review and editing, supervision, project administration, and funding acquisition. WastPan study group: resources and project administration. All authors contributed to the article and approved the submitted version.
