# Supplementary material for: Genomic epidemiology of nosocomial carbapenemase-producing Citrobacter freundii in sewerage systems in the Helsinki metropolitan area, Finland
Source: Front Microbiol. 2023 May 26;14:1165751. doi: 10.3389/fmicb.2023.1165751 (PMC10250652; doi:10.3389/fmicb.2023.1165751)
Supplement: Supplementary file 1 [file Table_1.DOCX]

*Supplementary Table 1. Isolate accession numbers and bioproject numbers for project PRJEB58690 at European Nucleotide Archive (ENA)* *(*https://www.ebi.ac.uk/ena/browser/view/PRJEB58690) *and* *sequencing statistics for all isolates (n=53) included in the study.*

|  |  |  | Sequencing statistics (Assembled) | | | | | |
| --- | --- | --- | --- | --- | --- | --- | --- | --- |
| Isolate ID | Accession number | BioSample | Contig Count | N50 | Read Count | Assembly Base Count | Average Contig Length | Average Coverage |
| 1 | ERS14413713 | SAMEA112302782 | 194 | 103413 | 3578096 | 5268342 | 27156 | 96 |
| 2 | ERS14413714 | SAMEA112302783 | 201 | 65785 | 2761818 | 5042458 | 25086 | 72 |
| 3 | ERS14413715 | SAMEA112302784 | 170 | 118410 | 3384946 | 5251841 | 30893 | 91 |
| 4 | ERS14413716 | SAMEA112302785 | 309 | 65963 | 2894732 | 5277167 | 17078 | 76 |
| 5 | ERS14413717 | SAMEA112302786 | 324 | 92388 | 3679396 | 5253687 | 16215 | 77 |
| 6 | ERS14413718 | SAMEA112302787 | 409 | 57805 | 3461026 | 5437661 | 13295 | 87 |
| 7 | ERS14413719 | SAMEA112302788 | 228 | 98366 | 3401690 | 5539460 | 24295 | 87 |
| 8 | ERS14413720 | SAMEA112302789 | 188 | 122784 | 3580944 | 5288063 | 28127 | 95 |
| 9 | ERS14413721 | SAMEA112302790 | 199 | 122718 | 3588422 | 5290588 | 26585 | 97 |
| 10 | ERS14413722 | SAMEA112302791 | 200 | 77005 | 2976002 | 5207941 | 26039 | 80 |
| 11 | ERS14413723 | SAMEA112302792 | 241 | 56990 | 2869978 | 5269224 | 21864 | 77 |
| 12 | ERS14413724 | SAMEA112302793 | 190 | 153736 | 4295458 | 5292771 | 27856 | 114 |
| 13 | ERS14413725 | SAMEA112302794 | 168 | 181734 | 4054700 | 5293916 | 31511 | 108 |
| 14 | ERS14413726 | SAMEA112302795 | 207 | 109086 | 3929872 | 5412226 | 26146 | 99 |
| 15 | ERS14413727 | SAMEA112302796 | 225 | 125514 | 4337486 | 5288077 | 23502 | 111 |
| 16 | ERS14413728 | SAMEA112302797 | 180 | 150176 | 4268418 | 5286021 | 29366 | 114 |
| 17 | ERS14413729 | SAMEA112302798 | 252 | 200251 | 3384100 | 5287022 | 20980 | 89 |
| 18 | ERS14413730 | SAMEA112302799 | 180 | 146719 | 3352560 | 5285527 | 29364 | 91 |
| 19 | ERS14413731 | SAMEA112302800 | 236 | 96926 | 3910904 | 5394358 | 22857 | 100 |
| 20 | ERS14413732 | SAMEA112302801 | 179 | 123249 | 3794686 | 5283617 | 29517 | 102 |
| 21 | ERS14413733 | SAMEA112302802 | 399 | 37268 | 1804556 | 5227784 | 13102 | 49 |
| 22 | ERS14413734 | SAMEA112302803 | 151 | 204525 | 4117692 | 5265322 | 34869 | 110 |
| 23 | ERS14413735 | SAMEA112302804 | 155 | 204537 | 4128282 | 5262902 | 33954 | 107 |
| 24 | ERS14413736 | SAMEA112302805 | 144 | 204537 | 4110690 | 5266599 | 36573 | 109 |
| 25 | ERS14413737 | SAMEA112302806 | 157 | 204530 | 4116218 | 5247865 | 33425 | 107 |
| 26 | ERS14413738 | SAMEA112302807 | 86 | 443977 | 4140086 | 5453623 | 63414 | 104 |
| 27 | ERS14413739 | SAMEA112302808 | 91 | 333950 | 4108178 | 5451695 | 59908 | 107 |
| 28 | ERS14413740 | SAMEA112302809 | 99 | 262702 | 4115300 | 5433236 | 54881 | 105 |
| 29 | ERS14413741 | SAMEA112302810 | 115 | 441377 | 4113316 | 5537119 | 48148 | 103 |
| 30 | ERS14413742 | SAMEA112302811 | 90 | 274015 | 4112388 | 5419240 | 60213 | 104 |
| 31 | ERS14413743 | SAMEA112302812 | 98 | 373460 | 4129656 | 5439795 | 55508 | 101 |
| 32 | ERS14413744 | SAMEA112302813 | 70 | 754600 | 4136542 | 5161813 | 73740 | 112 |
| 33 | ERS14413745 | SAMEA112302814 | 95 | 262746 | 4116980 | 5428022 | 57137 | 99 |
| 34 | ERS14413746 | SAMEA112302815 | 131 | 203293 | 4104188 | 5245153 | 40039 | 110 |
| 35 | ERS14413747 | SAMEA112302816 | 181 | 151352 | 4121352 | 5341903 | 29513 | 107 |
| 36 | ERS14413748 | SAMEA112302817 | 191 | 155308 | 4102548 | 5311388 | 27808 | 112 |
| 37 | ERS14413749 | SAMEA112302818 | 204 | 155304 | 4110702 | 5365818 | 26303 | 110 |
| 38 | ERS14413750 | SAMEA112302819 | 200 | 155360 | 4109192 | 5436547 | 27182 | 105 |
| 39 | ERS14413751 | SAMEA112302820 | 168 | 155332 | 4127864 | 5354396 | 31871 | 108 |
| 40 | ERS14413752 | SAMEA112302821 | 181 | 154584 | 4117888 | 5358627 | 29605 | 106 |
| 41 | ERS14413753 | SAMEA112302822 | 165 | 224532 | 4118458 | 5223793 | 31659 | 111 |
| 42 | ERS14413754 | SAMEA112302823 | 230 | 132667 | 4117822 | 5424370 | 23584 | 103 |
| 43 | ERS14413755 | SAMEA112302824 | 104 | 209300 | 4121848 | 5202874 | 50027 | 105 |
| 44 | ERS14413756 | SAMEA112302825 | 97 | 340543 | 4119492 | 5125397 | 52839 | 109 |
| 45 | ERS14413757 | SAMEA112302826 | 240 | 151356 | 4111628 | 5411029 | 22545 | 107 |
| 46 | ERS14413758 | SAMEA112302827 | 158 | 155340 | 4115518 | 5324317 | 33698 | 107 |
| 47 | ERS14413759 | SAMEA112302828 | 102 | 343803 | 4122250 | 5122301 | 50218 | 110 |
| 48 | ERS14413760 | SAMEA112302829 | 99 | 340701 | 4112350 | 5122253 | 51739 | 113 |
| 49 | ERS14413761 | SAMEA112302830 | 92 | 340543 | 4117082 | 5153032 | 56011 | 110 |
| 50 | ERS14413762 | SAMEA112302831 | 246 | 155284 | 4120940 | 5477672 | 22266 | 107 |
| 51 | ERS14413763 | SAMEA112302832 | 93 | 321971 | 4119488 | 5120819 | 55062 | 111 |
| 52 | ERS14413764 | SAMEA112302833 | 185 | 154108 | 4116546 | 5386622 | 29116 | 106 |
| 53 | ERS14413765 | SAMEA112302834 | 166 | 164359 | 4115320 | 5325039 | 32078 | 108 |
