# Supplementary figures and images for: Genomic epidemiology of nosocomial carbapenemase-producing Citrobacter freundii in sewerage systems in the Helsinki metropolitan area, Finland
Source: Front Microbiol. 2023 May 26;14:1165751. doi: 10.3389/fmicb.2023.1165751 (PMC10250652; doi:10.3389/fmicb.2023.1165751)

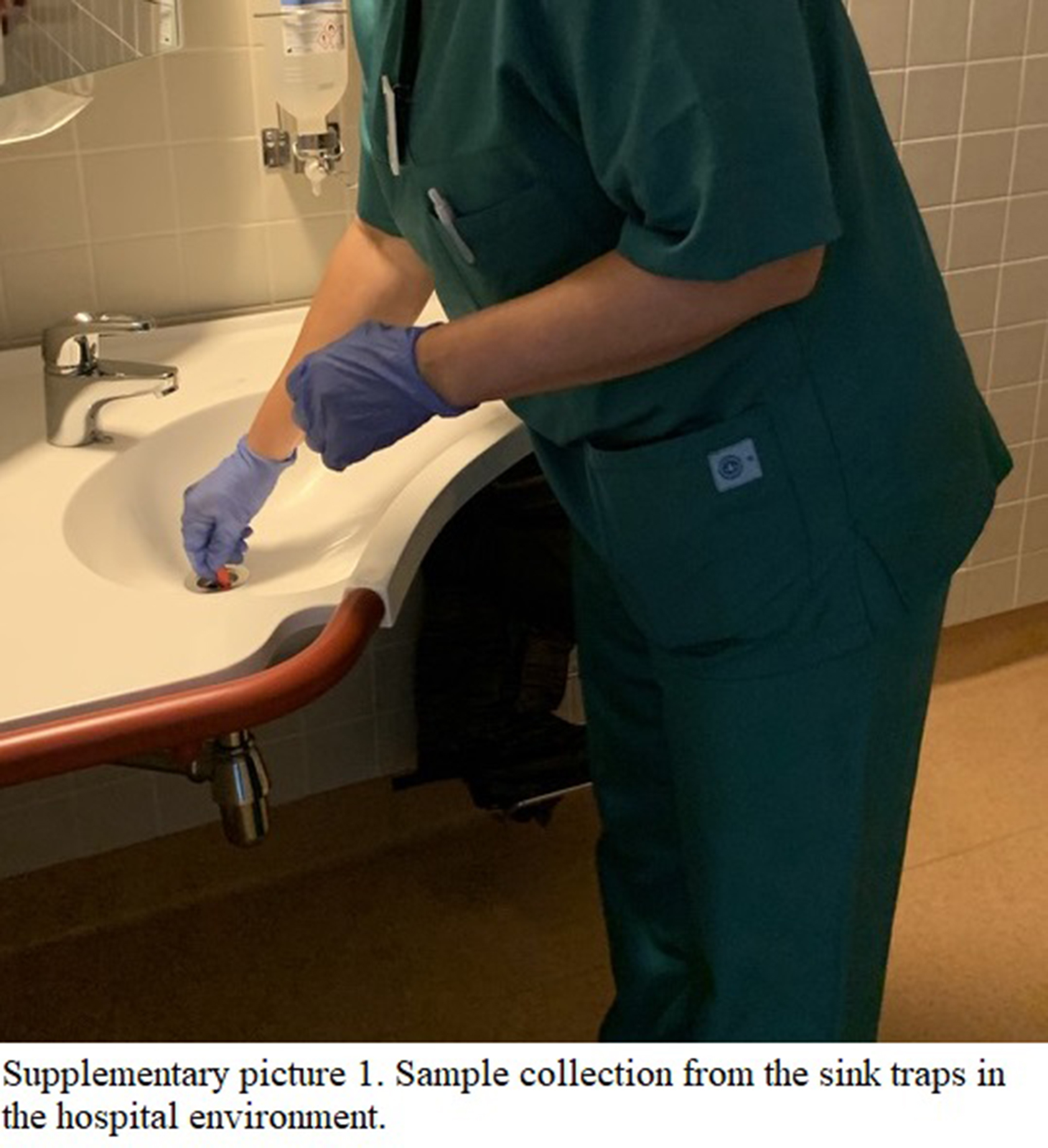

Supplement: Supplementary file 2 [file Image_1.JPEG]

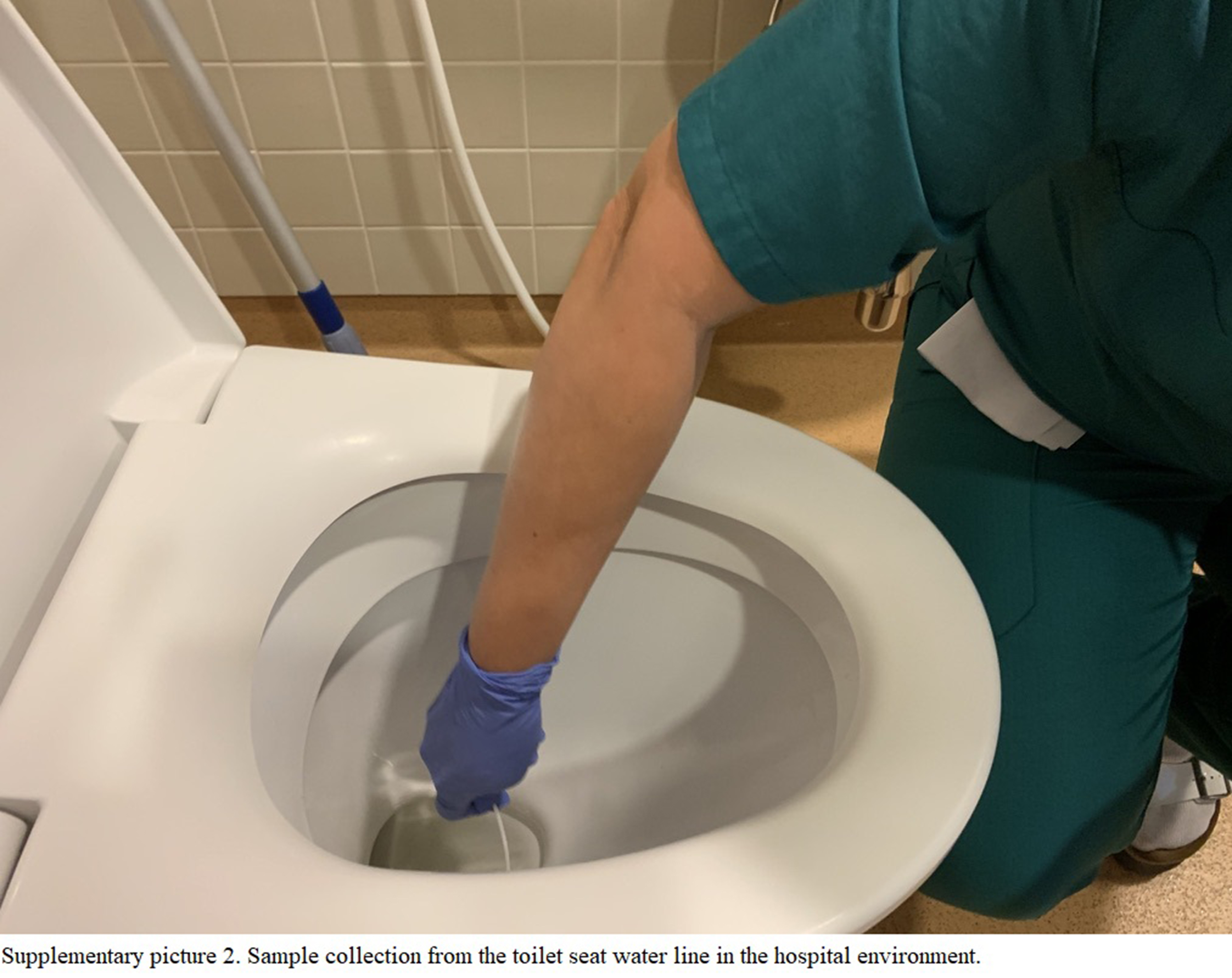

Supplement: Supplementary file 3 [file Image_2.JPEG]
